# Training early career researchers to use meta-research to improve science: A participant-guided “learn by doing” approach

**DOI:** 10.1371/journal.pbio.3001073

**Published:** 2021-02-24

**Authors:** Tracey L. Weissgerber

**Affiliations:** QUEST–Quality | Ethics | Open Science | Translation, Charité –Universitätsmedizin Berlin, Berlin Institute of Health (BIH), Berlin, Germany

## Abstract

This Community Page article describes a participant-guided "learn by doing" initiative, in which a multidisciplinary, global team of early career researchers learned meta-research skills by working together to design, conduct and publish a meta-research (science of science) study. Participants can now apply these skills to solve problems in their own fields.

## Teaser

Meta-research, or the science of science, is a powerful technique that scientists can use to improve science, however most scientists are unaware that meta-research exists and courses are rare. This initiative demonstrates the feasibility of a participant-guided “learn by doing” approach, in which a multidisciplinary, global team of early career researchers learned meta-research skills by working together to design, conduct and publish a meta-research study.

## Introduction

The reproducibility crisis has highlighted widespread issues with experimental design and methods [1–3], statistical analysis and reporting [4–6], and data visualization [7]. Meta-research, or the science of science, is a powerful technique that scientists can use to address these problems [8]. Meta-research is different from a meta-analysis, in which researchers combine all studies addressing a research question to estimate the size of the effect. Meta-researchers apply the scientific method to study science itself [8]. Data on the frequency of problematic vs. good scientific practices allows researchers to develop and test targeted solutions. Meta-research may also examine topics like hiring practices, journal guidelines, and funding agency policies. In addition to encouraging authors to adopt more transparent and reproducible practices, meta-research can contribute to policy change. Our meta-research paper on the inappropriate use of bar graphs to present continuous data [7], for example, encouraged many journals to adopt policies requiring authors to replace bar graphs with more informative graphics [9,10].

Most scientists are unaware of meta-research and few are trained to conduct these studies. Courses in this relatively new field are rare. This paper provides an overview of the eLife Ambassadors Meta-research Team, in which a multidisciplinary, global team of early career researchers learned meta-research skills by working together to design, conduct and publish a meta-research study. The participant-guided, “learn by doing” approach provided hands on experience in using meta-research to understand and solve problems with scientific research. The teams’ research examined the legibility and interpretability of images in scientific publications [11]. Participants systematically assessed image-based figures among papers published in the top 15 journals in three fields; plant sciences, cell biology and physiology. The paper includes visualizations that illustrate solutions to common problems, including missing scale bars, misplaced or poorly marked insets, images or labels that were inaccessible to colorblind readers, and insufficient explanations of colors, labels or annotations. Participants can apply their new skills to improve research practices in their respective fields.

The “learn by doing” approach has many advantages. Whereas students in traditional courses complete assignments and exams, participants in “learn by doing” courses collaborate to create something of value to the scientific community. The hands-on approach forces participants to apply the concepts discussed in virtual meetings. The opportunity to complete a research study is popular with participants and the resulting publication gives participants credit for their work. Finally, participants gain skills for collaborating in multidisciplinary teams. This approach, however, introduces unique challenges. A participant-guided, “learn by doing” course is best described as controlled chaos. Instructors must constantly adapt to changing circumstances. Unknowns at the beginning of the course include participants’ expertise and personalities and potential projects. These uncertainties are gradually reduced as participants get to know each other, select a project and finalize the study design and protocol. This paper provides a brief overview of this virtual participant-guided, “learn by doing” initiative and presents strategies for running a successful “learn by doing” course.

### A participant-guided, “Learn by Doing” approach

This initiative took place within the eLife Ambassadors Program, which brings together early career researchers who want to improve scientific publishing and research culture. More than 200 early career researchers from around the world participate in the program each cycle, including graduate and medical students, postdoctoral fellows and new principal investigators. Ambassadors can join initiatives focused on many topics, such as preprints, reproducibility, and meta-research.

Eighteen ambassadors joined the Meta-research Team. This virtual team included scientists at different institutions in eight countries (US, UK, Germany, Italy, Portugal, Egypt, Japan, and India). Participants came from many fields, including developmental biology, plant sciences, hematology, and cancer biology. Online discussions via Slack and Discourse were used to resolve simple questions and communicate asynchronously across time zones. Videoconference calls focused on in-depth discussions about complex topics. At the beginning of each phase (Box 1), the instructor provided an overview of concepts, tasks and tools needed for that phase. Subsequent sessions consisted of instructor-moderated discussions and group problem solving. Participants posed questions or described challenges that they were experiencing. Team members explored possible solutions and selected the best approach. Topics were not planned in advance, as they were often specific to the project and were framed around students’ activities during the previous weeks. A condensed version of this approach is being used to teach a six-month credited course for fifteen graduate students in different fields at four Berlin universities. Whereas the Meta-research Team was open to any ambassador, students had to apply for the Berlin course.

This participant-guided, “learn by doing” approach has limitations. A single project is insufficient to learn the complex skills required to conduct any meta-research study. This initiative focused on a literature survey or systematic review style meta-research study. In this common, basic study design, researchers systematically assess a set of records based on pre-defined criteria. The initiative’s long-term effects are presently unknown. Even if participants do not conduct future meta-research studies, they have gained valuable skills for systematically evaluating the strengths and weaknesses of research in their own fields. Participants may use these skills to encourage their colleagues to adopt more transparent and reproducible practices.

**Box 1. Program phases**

Participants from the 2018 Ambassadors Meta-research Team proposed and designed the study, developed screening and abstraction protocols, and screened papers to identify eligible articles (1 to 4, beginning of 5). Participants from the 2019–2020 Ambassadors Meta-research Team refined the data abstraction protocol, completed data abstraction and analysis, prepared the figures and manuscript and provided feedback on the manuscript (5 and 6). As this was a volunteer initiative, there were prolonged breaks when most participants were busy with graduate program requirements or deadlines. In the condensed Berlin course, students complete all phases of the project.

1. **Introduction to meta-research:** Participants read meta-research articles to learn common methods for a literature survey or systematic review style meta-research study. The group shared their knowledge by writing a “Science of science reading list for peer reviewers” blog post.
2. **Project development:** Each participant developed a meta-research project proposal and some participants conducted feasibility tests. During calls, participants shared ideas and study designs and got feedback from their peers. Participants discussed the advantages and disadvantages of alternative designs and other possible solutions. This hands-on problem solving trained participants to recognize and fix common design problems.
3. **Project selection:** Two proposals were feasible and scientifically sound. The group selected one proposal to complete as a team.
4. **Protocol development:** Participants worked in small teams to prepare and test screening and data abstraction protocols. The screening protocol determines which articles will be included in the study, whereas the abstraction protocol specifies which data will be collected on each article.
5. **Conducting the study:** Journal and article screening, data abstraction training, data abstraction and data analysis were performed as described in the study methods [11].
6. **Manuscript preparation:** Participants attended a training session on designing communication and dissemination strategies. Concepts discussed in this session were used to design figures, prepare the manuscript and create teaching slides. Participants shared the abstraction protocol, data, code and teaching slides in a public repository [12].

### Lessons learned

This section outlines strategies for running a successful “learn by doing” course.

#### Create a positive and inclusive group dynamic

Participants must collaborate to complete the project; therefore it’s important to quickly get everyone working towards a common goal. This includes agreement on the objectives, process and course deliverables before the project is known. Team building strategies used in this initiative were described previously. The instructor should moderate calls to ensure a balanced discussion and fill in knowledge gaps once all participants have spoken. While knowledge gaps will be common in early sessions, the group should transition to participant-driven discussions with few inputs from the instructor over time.

#### Create opportunities for participants to get to know each other

In person meetings were impossible for this global virtual team. Strategies for connecting team members included introductions or brief personal updates on each call. Many tasks were performed in groups of 2–5 people, providing opportunities for personal conversations. We created a text-messaging group in 2020 to provide support during COVID-19 pandemic lockdowns. Social texts continued after lockdowns ended. Many participants reported that the supportive team and relationships with others kept them engaged.

#### Have participants select a project that is interesting to them

The group selected the project, which was proposed by a participant, after three months of constructive criticism, group problem solving, and feasibility testing. This process was designed to build consensus around the strongest projects. This was a multidisciplinary team; therefore the fact that many fields use images was a distinct advantage of the selected proposal. Everyone could contribute and many participants used images in their own research.

Teaching participants to design studies in an unfamiliar field is very time consuming. Some proposals may require extensive design changes; others may not be feasible. In the condensed follow-up course, the instructor proposed two projects and participants could propose a “wild-card” project. This shortened proposal development, while allowing participants to consider strong proposals from their classmates. The instructor’s projects were not fully developed proposals, allowing students to work through the study design process.

#### Focus on why

Participants make many decisions while designing and conducting a study. While individual decisions may be highly technical and topic specific, over time students become adept in applying concepts and evaluating trade offs between possible solutions. Focusing on why the group is choosing a particular option builds reasoning skills and enhances future decision-making.

#### Use participants’ unique skills to enhance the project

Participants came from many disciplines and had a wide range of skills. This diverse expertise strengthened the project. The team assessed three fields to improve generalizability. Those with coding experience ran the literature search, managed databases, and analyzed data. Visualization experts prepared figures illustrating common mistakes and recommended practices.

#### Use unexpected events as teaching opportunities

Unanticipated problems occur in every study. Instructors can use these events to teach problem solving skills. One of the journals included in the sample, for example, was de-indexed during the project. The team identified three possible solutions and discussed the advantages and disadvantages of each before deciding how to proceed. Participants applied the concepts that they learned to solve subsequent problems.

#### Use regular meetings and progress updates to maintain momentum

Regular progress motivates and energizes participants. Participants preferred to meet every two to four weeks during active periods. The condensed course met weekly. Small groups shared their progress with everyone in the “updates” channel.

#### Share interim outputs

Interim outputs, like the “Science of science reading list for peer reviewers”, motivate participants and encourage them to discuss their work. Some participants presented the groups’ activities at institutional research days. The group presented quarterly updates on ambassador calls and in newsletters. The Berlin students presented their work at a virtual meta-research conference.

#### Build a diverse team to expand participants’ horizons

This was the first time that most participants had worked in a global, multidisciplinary team. Participants identified problems that were common to many fields, while exploring factors that were unique to their country or discipline. This emphasized the need for systemic improvements and the complexity of implementing changes across the global scientific community.

#### Provide leadership opportunities

Small group activities provided many opportunities for participants to develop leadership and teamwork skills, including leading small teams, chairing calls, planning and coordinating tasks, giving regular progress updates, conflict resolution and consensus building.

## Conclusion

This initiative demonstrates the feasibility of a participant-guided “learn by doing” approach, in which a multidisciplinary, global team of early career researchers worked together to design, conduct and publish a meta-research study. The strategies outlined here may be valuable to others who want to use “learn by doing” approaches to teach new skills or design courses in which students collaborate to create something of value to a broader community. In addition to the course material, participants learn skills for multidisciplinary team science.
